# The effect of performing versus preparing a task on the subsequent switch cost

**DOI:** 10.1007/s00426-019-01254-7

**Published:** 2019-10-17

**Authors:** Rachel Swainson, Laura Prosser, Kostadin Karavasilev, Aleksandra Romanczuk

**Affiliations:** School of Psychology, University of Aberdeen, William Guild Building, Aberdeen, AB24 3FX Scotland, UK

## Abstract

Behaviour occurs not as isolated incidents, but within an ongoing sequence of events. The task-switching paradigm provides a useful way to investigate the impact of different events upon subsequent performance. An implication of two-stage task-switching models is that preparing a task without performing it might affect task readiness only to a limited extent. However, recent research has surprisingly shown larger switch costs following preparation (“cue-only” trials) than following performance (“completed” trials). We set out to conduct a rigorous comparison of the size of switch costs following cue-only versus completed trials. In Experiments 1 and 2, we controlled the timing between critical trial events. This had the effect of roughly equating, but not reversing, the relative size of switch costs. In Experiment 3, we restructured the paradigm to equate the predictability of cue and target events. Switch costs following cue-only trials were now smaller than those following completed trials. These studies confirm that task preparation alone is sufficient to drive subsequent switch costs. They also indicate that task performance might increase the size of these costs, consistent with two-stage task-switching models. Switch costs appear to be affected by both the timing and predictability of trial events.

## Introduction

Behaviour occurs within an ongoing sequence of events rather than as individual, isolated events. Therefore, it makes sense to study the cognitive control of behaviour in the light of preceding events. Such events will include covert mental processes such as plans and intentions that were not necessarily carried out, as well as our own previous overt behaviour. In this paper, we aim specifically to assess the relative impact of preparation to perform a task versus actual performance of that prepared task upon subsequent performance. In other words, we ask whether what we “know” (prepare, intend) has as much of an impact as what we “do” (perform, enact) upon what we do next.

The relative effects of “knowing” and “doing” have long been of interest in the psychological literature (see the recent review and theoretical model by Brass, Liefooghe, Braem, and De Houwer, 2017), where a number of examples indicate that the impact of what we prepare or intend to do can be relatively weak compared with the impact of what we actually do. Patients with frontal lobe lesions have been observed to perseverate to a previously relevant rule despite being able to articulate the correct rule (Milner, 1963). Healthy individuals can show that they are able to understand and remember a task rule, but then fail to implement it when required (goal neglect: Duncan, Burgess, and Emslie, 1995). Intentions have been shown to exert less of an interference effect upon ongoing behaviour than that produced by actions (Waszak, Wenke, and Brass, 2008). However, doing might not always be more powerful than knowing: intended but unperformed actions can prove to be more persistent than those that were executed (Bugg and Scullin, 2013).

The experimental task-switching paradigm is designed to assess the effects of performing a task upon our subsequent ability to perform either the same task or a different task (see reviews by Kiesel et al., 2010; Monsell, 2003; and Vandierendonck, Liefooghe, and Verbruggen, 2010). The “switch cost” effect that it generates (i.e., poorer performance for switching than repeating tasks) provides evidence that the task that was relevant on the previous trial affects the speed and accuracy of current performance. Recently, task-switching researchers have begun to ask whether the switch cost is driven by what task was prepared on the preceding trial (what was “known”), or what task was actually performed (what was “done”). These studies modified the basic paradigm such that on a proportion of trials some of the usual stages of task-processing are missing. It can then be asked whether the remaining stages are sufficient to generate a switch cost on the subsequent trial.

Schuch and Koch (2003) ran such a study, involving switching between different number-judgement tasks (odd/even and smaller/larger judgements). They eliminated the later stages of task-processing on a proportion of trials by converting them into “no-go” trials. On these trials, a no-go signal (either a low tone presented concurrently with the target stimulus or a stimulus upon which neither task could be performed) indicated that the prepared task should not be performed. The authors found that no RT switch cost was present on trials immediately following no-go trials. It was concluded that the response-selection stage of task performance is necessary to cause a task to persist and cause a subsequent switch cost and that preparation alone is insufficient to do so. A number of other studies have also shown that switch costs are absent following no-go trials (e.g. Astle, Jackson and Swainson, 2006; Lenartowicz, Yeung and Cohen, 2011; Los and Van der Burg, 2010; Verbruggen, Liefooghe, Szmalec, and Vandierendonck, 2005; Verbruggen, Liefooghe, and Vandierendonck, 2006).

The apparent importance of task performance in driving the subsequent switch cost seen in these no-go studies is in line with some, but not all, models of task-switching. It fits well with the task-set inertia and task-set priming accounts of Allport and colleagues (e.g., Allport, Styles, and Hsieh, 1994; Allport and Wylie, 2000), according to which performance of a task leads to relative facilitation of performance on subsequent task-repeat trials and interference on subsequent task-switch trials. While top–down control determines which task is performed in those accounts, a switch between intended tasks is not seen to contribute substantially to the switch cost measure itself. The “two-stage” task-switching models (e.g. Meiran, 2000; Rogers and Monsell, 1995; Rubinstein, Meyer and Evans, 2001), which include both performance-driven and preparatory task-set reconfiguration processes, also seem to predict that performance should drive at least a part of the subsequent switch cost that could not have been driven by preparation alone. The same seems to be true of the memory-based model of Oberauer, Souza, Druey and Gade (2013), according to which the switch cost includes time taken both by preparatory task-set reconfiguration and by overcoming interference driven by previous performance. In contrast, the Cognitive Control Model of Altmann and Gray (2008) has priming of cue identification as the source of the RT switch cost, and includes no special role for task performance. The compound-cue model of Schneider and Logan (2005), which posits no involvement of “tasks” as such, also puts the source of the measured switch cost at the stage of cue processing. Neither of these latter two models therefore would seem to predict a specific role for performance in driving an increased subsequent switch cost.

Lenartowicz, Yeung, and Cohen (2011) pointed out that the absence of any switch cost at all following no-go trials on which task preparation preceded the no-go stimulus (as in Schuch and Koch, 2003) does not seem to fit well with the finding that preparation can reduce the switch cost on the current trial. One would expect preparation on no-go trials to generate a switch cost measurable on subsequent trials with short preparation intervals, even if response selection was necessary to make the cost persist at long subsequent preparation intervals (the so-called “residual” switch cost). Lenartowicz et al. (2011) hypothesised that no-go signals might interfere with the effects of prior preparation, abolishing its potential to generate a subsequent switch cost. In their Experiment 2, they replaced no-go trials with “cue-only” trials, on which only a task cue was presented. Following a preparation interval, the trial ended with neither a target stimulus nor a no-go stimulus being shown. Using this method, Lenartowicz et al. found a substantial switch cost following preparation alone. That result was replicated by Swainson, Martin and Prosser (2017; see also similar results using different designs by Brass and von Cramon, 2002, and Desmet, Fiat, and Brass, 2012).

These cue-only studies show that preparation can produce a subsequent switch cost, in line with the idea that task preparation can drive at least part of the task-switching process. However, a new puzzling feature was evident in the data from both the Lenartowicz et al. (2011, Expt. 2) and Swainson et al. (2017, Expt. 2) studies. That is, the switch cost measured on trials with short preparation intervals following cue-only trials was approximately twice as large as that following “completed” trials (i.e., standard trials, where a target stimulus was presented after the preparation interval and an appropriate response executed). This pattern in the data was not predicted in either study (and was not tested for significance in Lenartowicz et al., 2011). But it may be important, because it does not appear to sit easily alongside either performance-based or two-stage task-switching models, according to which task preparation plus task performance should produce a more substantial subsequent switch cost than task preparation alone. Instead, it seems to fit rather better with preparation-based models such as that of Altmann and Gray (2008), as well as with the finding that unperformed prospective intentions can persist more stubbornly than those that have been performed (Bugg and Scullin, 2013). Hence, we aimed to establish what might account for the effect and whether it could be eliminated or even reversed if we removed potential confounds.

As well as comparing the size of switch costs at the shortest preparation interval, we wished to obtain a further measure for comparison, one that might tell us something about the nature of the switch cost rather than simply its size. We measured the RISC (“reduction in switch cost”) effect (Monsell and Mizon, 2006; also termed the “preparation effect”, Rogers and Monsell, 1995), which indicates the extent to which an existing switch cost can be reduced during the preparation interval of the current trial. We hypothesised that a cost driven only by preparation on the preceding trial might be more easily overcome during preparation on the current trial than would a cost that followed task performance. If so, the cost following preparation should exhibit a larger RISC effect than would the cost following performance. Such a finding would fit the proposed separation of endogenous and exogenous processes in two-stage task-switching models, as well as being in line with other proposals that emphasise the role of performance over that of preparation (e.g. Allport and Wylie, 2000).

Across the three experiments, we progressively addressed a number of issues with previous studies, as follows. In Experiment 1, we addressed a possible bias associated with two types of between-trial interval: the response-cue interval and the cue-cue interval. We reasoned that differences in these intervals between conditions might have allowed the effective strength of a task to diminish more by the end of completed trials than by the end of cue-only trials, plausibly reducing the relative size of the switch cost following completed trials. In Experiment 2, we aimed to prevent any part of the switch cost being “prepared away” before it could be measured, by using a 0 ms (instead of 300 ms) preparation interval. In Experiment 3, we addressed the possibility that participants might have been more ready to switch following completed than cue-only trials, potentially lowering the switch costs following completed trials. To do this, we changed the structure of trials such that there was an equal likelihood of the next “stimulus event” being a cue or a target.

## Experiment 1

This experiment eliminated an imbalance in two aspects of between-trial timing. First, the substantial response-cue interval was removed. In Swainson et al. (2017, Expt. 2), the response-cue interval used before short preparation trials was 1300 ms; in Lenartowicz et al. (2011, Expt. 2), it was 1500 ms. These intervals provided a potential opportunity for the task used on completed trials to be discarded before the subsequent trial, therefore potentially reducing the switch cost. No such opportunity would have been present following cue-only trials. Second, we controlled the cue-cue intervals. Cue-only trials had previously always been shorter than completed trials (when preparation intervals were matched) and as a result, substantially more time elapsed between the preceding cue and the current cue when the preceding trial was a completed trial than when it was a cue-only trial. (This difference was approximately 2000 ms when preparation interval on the current trial was short, partly due to the presence of a response-cue interval already described, but also partly due to completed trials including target and response events that do not occur on cue-only trials). It is quite feasible that a lengthened cue-cue interval would tend to decrease the subsequent switch cost (as noted by Lenartowicz et al., 2011), because of the decay of cue-based effects. Indeed, Altmann and Gray’s (2008) model of task-switching posits that access to the task code generated by processing the previous cue is critical to the size of switch costs measured on the current trial. We could not match cue-cue intervals exactly across conditions, since RTs are inherently variable (and we wished to avoid equating intervals using a response-cue interval, for reasons explained above). Instead, our solution partially reversed the previous bias rather than removing it, as follows. We added a new, longer (2400 ms) preparation interval into the design, which occurred unpredictably in place of half of the previous “long” (1000 ms) preparation intervals on both cue-only and completed trials. We then analysed trials preceded by either a cue-only trial with 2400 ms preparation or a completed trial with 1000 ms preparation (plus an upper RT limit of 1400 ms). Hence, cue-cue interval could now never be longer following completed trials than it was following cue-only trials.

The key question in this experiment was whether the switch cost (measured on trials with a short preparation interval) following completed trials would now be larger than that following cue-only trials or whether the opposite pattern, seen previously, would remain. As a part of this analysis, we also tested whether each of the switch costs (i.e., following cue-only and following completed trials) at the short preparation interval would themselves be statistically significant. In addition, we tested whether an increasing preparation interval on the current trial would enable the switch cost following cue-only trials to be more rapidly overcome (i.e., to show a larger RISC effect) than that following completed trials, again controlling for cue-cue interval. Finally, we tested directly whether increasing preparation time on the preceding trial led to decreased switch costs, since this would support the idea that unbalanced cue-cue intervals had caused differing switch costs in previous studies.

## Method

### Participants

Forty-one participants (38 female, 3 male) were tested in return for course credit. The age range was 18–34 years (median 19 years).

### Materials

The study was run on PCs running E-Prime 2.0 software (Psychology Software Tools, Inc., http://www.pstnet.com), with button–boxes for responses (Cedrus Corporation, 2003; Psychology Software Tools). Participants used their left and right index fingers to press horizontally adjacent buttons. Each target stimulus consisted of a coloured shape (2.5 cm wide) presented centrally on a black background: a circle, star, triangle or square coloured red, blue, yellow or green. For each task there were two alternative task-cueing words (presented in white, Courier New size 40 font, centrally on a black background): “COLOUR” and “HUE” cued the colour task; “SHAPE” and “FORM” cued the shape task. Cues switched on every trial to avoid confounding cue-switching with task-switching. Stimulus features (e.g. “red” and “star”) for the current trial (*n*) never matched those of the most recently presented target (trial *n* − 1 following completed trials; trial *n* − 2 following cue-only trials) to ensure that any switch costs found would be at task-level rather than feature-level. The response for each trial was selected first (at random) and then an appropriate stimulus feature mapping to that response was allocated (again, at random). This was done independently for each stimulus dimension (colour and shape), so response congruity was unbiased.

### Procedure

Participants were each tested in a single testing session, each approximately 105 min long. Up to four participants were tested concurrently in a small room separated into “booths” by screens so that no participant could see any other participant’s responses or their monitor. Participants were assigned left-index-finger responses for two colours and two shapes and right-index-finger responses for the remaining two colours and two shapes. (There were 36 possible combinations of these mappings, assigned consecutively to participants as each was tested. Since participants were excluded without reference to their assigned mappings, and only 32 participants remained following exclusions, the 36 combinations were not equally represented in the analysed dataset. Instead, data from zero, one or two participants with each of the 36 possible mappings were included in the analysis.) These mappings were presented on-screen as a reminder before every block of trials. There were four blocks of practice trials in total. First, there were 20 trials of the colour task only; then, 20 trials of the shape task only; then, 20 trials with both tasks randomly intermixed. In these first three blocks, all trials were completed trials, so they included presentation of a task-cue and a target stimulus and they required a button-press response. The preparation intervals (cue onset to target onset) in these blocks matched those used for the completed trials in experimental blocks: 50% at 300 ms, 25% at 1000 ms, and 25% at 2400 ms. The final practice block of 20 trials introduced cue-only trials, in the same proportion as in the experimental blocks, as described below; on these trials a task-cue but no target stimulus was presented. Before this block, participants were informed that on some trials no target would be presented and that every target should only be processed according to the cue that immediately preceded it. At the end of the practice blocks, participants were given the option to repeat practice, if they were not confident of the rules; otherwise they proceeded to the experimental blocks.

There were 32 blocks of experimental trials, each block 56 trials long, with a break after each block that ended when the participant was ready to continue. After every eight blocks, participants were encouraged to take a longer break than usual, if they wished to. Participants were asked to respond quickly and accurately and to try to use the cue to improve performance by preparing for the appropriate task.

The trial-type on each trial was selected at random according to a set probability (with the restriction that cue-only trials could not occur consecutively), such that the overall proportions would approximate the following values. In terms of task transition: 50% trials involved a task-switch; 50% trials a task-repeat. For consistency with our previous study (Expt. 2 of Swainson et al., 2017), 30% of trials with a substantial preparation interval (here, either 1000 ms or 2400 ms) were cue-only and the remaining 70% of those trials were completed. Completed trials with a short preparation interval were also included, the proportion of these being equal to that of completed trials with a long preparation interval. Hence, the relative proportions of the different trial-types (in terms of trial completion and preparation interval) out of a total of 170 were as follows: 15/170 cue-only_1000ms_; 15/170 cue-only_2400ms_; 35/170 completed_1000ms_; 35/170 completed_2400ms_; 70/170 completed_300ms_. (N.B., there were no cue-only trials with a preparation interval of 300 ms.)

**Fig. 1:**
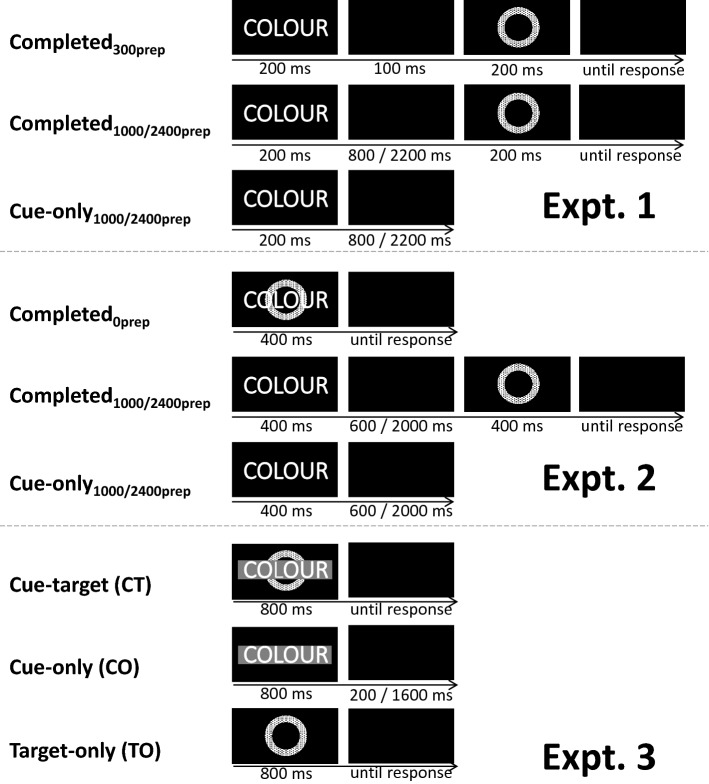
Timing of trial events in each of the trial-types in Expts. 1–3. Images are not to scale. Dotted fill pattern for circle targets shown here represents a solid colour used in the experiment (see methods)

Figure 1 shows the timings of trial events. All trials began with the presentation of a cue for 200 ms and then a blank screen for 100, 800 or 2200 ms: this produced the three preparation intervals of 300, 1000 and 2400 ms. Cue-only trials ended after the preparation interval and were always followed immediately by a completed trial, with no additional inter-trial interval occurring. Completed trials, however, continued after the preparation interval with the presentation of a target stimulus for 200 ms and then a blank screen until a response was recorded; RTs were timed from the onset of the target. The next trial (either cue-only or completed) began upon detection of the response. Although we had intended there to be no response-cue interval at all, a short, variable delay (below 50 ms on 99% trials; mean 30 ms) was found to have occurred, while the software processed a response and began the next trial; this delay did not change across experimental conditions. On completed trials where the wrong response was made, “INCORRECT” was presented in magenta for 500 ms, followed by a 500 ms blank, before the next trial began.

### Data processing

Data were processed before analysis to exclude particular trials and participants, as follows. Trials with RTs below 200 ms or above 2000 ms (as in Swainson et al., 2017), trials following an incorrect response and the first trial of each block were excluded. Trials with an incorrect response were excluded from the analysis of RTs. Participants who scored less than 70% correct overall on completed trials in experimental (non-practice) blocks were excluded (six participants); among the remaining participants, those with more than 10% trials removed for being too fast or too slow were also excluded (three participants). A mean of 4% trials (range 0–9%) across participants were excluded from the final dataset for being too fast or too slow. No limit was set for the number of trials per participant available for each analysis condition, but post hoc analysis of trial numbers showed that in all cases at least 20 trials were included per analysed condition for each participant.

We controlled for cue-cue interval as follows. Where the preceding trial was a completed trial, it had to have had a preparation interval of 1000 ms and an RT no longer than 1400 ms; i.e., total trial duration maximum 2400 ms. (Across participants, a mean of 11% trials, range 0–27%, were discarded to meet the RT criterion applying to the previous trial.) Where the preceding trial was cue-only, it had to have had a preparation interval of 2400 ms. This meant that the cue-cue interval was never longer following completed than following cue-only trials. It is important to note that this procedure actually *over*-corrects the imbalance that was present in the previous studies (Lenartowicz et al., 2011; Swainson et al., 2017) rather than matching it across conditions.

### Design and analysis

The design was entirely within-subjects. Dependent variables were RT and percentage errors.

Analysis 1 dealt with data from trials with a short (300 ms) preparation interval only, controlling for cue-cue interval as described above. The presence of switch costs on these trials was tested for separately according to whether the trials followed completed trials or cue-only trials, using paired-samples *t* tests (switch versus repeat). Then the relative size of switch costs at this short preparation interval (300 ms) was assessed by repeated-measures ANOVAs with the factors preceding trial completion (completed, cue-only) and transition (repeat, switch), whereby a significant interaction would show that whether a trial was only prepared (cue-only) or also performed (completed) affected the size of the subsequent switch cost.

Analysis 2 tested whether there was any difference in the rate of reduction of the switch cost with increasing preparation interval on the current trial (i.e., the RISC effect), again on data that controlled for cue-cue interval. Hence, this analysis included performance data from trials with a substantial preparation interval as well as from trials with a short preparation interval. The 1000 ms and 2400 ms preparation intervals (applying to the current trial) were treated as a single condition in this analysis in order that sufficient trials would be available per analysed condition; we expected that both of these intervals would allow sufficient time for preparation to occur on the current trial. A repeated-measures ANOVA was run with three factors: current preparation interval (300 ms, [1000 or 2400 ms combined]), preceding trial completion (completed, cue-only) and transition (repeat, switch). A significant three-way interaction would indicate that the size of the RISC effect differed according to whether the previous trial had been only prepared or also performed.

Analysis 3 looked for evidence that cue-cue interval might affect the size of switch costs at the short preparation interval since we had hypothesised that a difference in cue-cue interval across conditions might have been one of the causes of the unexpectedly large switch costs following cue-only trials in earlier studies. Preceding preparation interval (1000, 2400 ms) was used as the relevant factor here, with preceding trial completion (completed, cue-only) as the second factor, and switch cost (switch cost = [switch – repeat]) as the dependent variable. Where the preceding trial was a cue-only trial, the preparation interval on that trial was directly related to the cue-cue interval between the preceding and current trials. Where the preceding trial was a completed trial, the preparation interval on that trial would also tend to affect the cue-cue interval, although less directly because the variable RT present on those trials would also affect the cue-cue interval. There was no control of cue-cue interval in this analysis, and therefore no restriction was placed on the length of RT on preceding completed trials.

Where non-significant effects were to be used to support key conclusions (specifically, conclusions regarding switch cost comparisons between preceding trial completion conditions), we supplemented the above analyses with JZS Bayesian t-tests (Rouder, Speckman, Sun, Morey, and Iverson, 2009), using the BayesFactor package in R (t-testBF; Morey and Rouder, 2018). The value of the Bayes Factor (BF_10_) reflects the strength of evidence for the alternative hypothesis relative to that for the null hypothesis, with values below 1 favouring the null. A BF_10_ of 0.2, for instance, would indicate that the null hypothesis was five (1/0.2) times as likely as the alternative hypothesis, given the data.

## Results

**Fig. 2:**
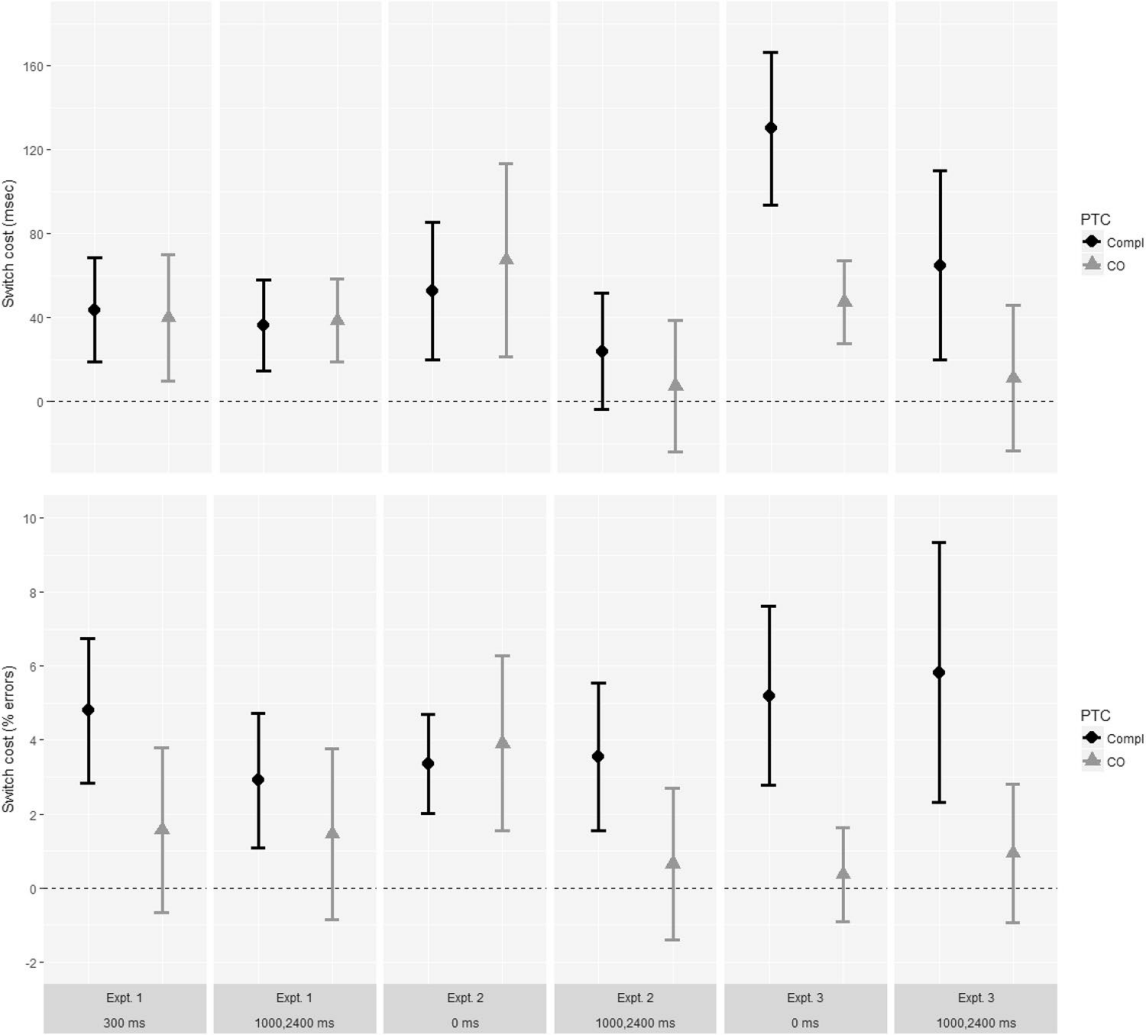
Switch costs ([switch – repeat] performance, in terms of mean RT and mean % errors) in Expts. 1–3, according to current preparation interval (left, 300 or 0 ms; right, combined across 1000 and 2400 ms), and preceding trial completion (PTC: Compl = completed; CO = cue-only). These data are controlled for cue-cue interval as described in the text and are relevant to Analyses 1 and 2 in each experiment. Error bars show 95% confidence intervals. For data on repeat and switch trials separately, see Table 1

Data relevant to Analyses 1 and 2 are shown in Table 1 and Fig. 2. Data relevant to Analysis 3 are shown in Table 2.

**Table 1:** Means (M) and standard deviations (SD) of performance and switch cost scores (RT and % errors) in Experiments 1–3, according to current preparation interval and preceding trial completion

Experiment/preparation interval	Preceding trial completion	Repeat		Switch		Switch cost	
		M	SD	M	SD	M	SD
RT (ms)							
Experiment 1							
300 ms	Completed	824	100	868	109	44	69
	*Cue*-*only*	*887*	*147*	*927*	*133*	*40*	*84*
1000, 2400 ms	Completed	828	122	865	118	36	60
	*Cue*-*only*	*798*	*118*	*836*	*116*	*39*	*55*
Experiment 2							
0 ms	Completed	1017	186	1070	193	53	96
	*Cue*-*only*	*1159*	*219*	*1227*	*221*	*67*	*136*
1000, 2400 ms	Completed	884	138	908	156	24	82
	*Cue*-*only*	*885*	*163*	*892*	*149*	*7*	*92*
Experiment 3							
0 ms	Completed	826	128	956	155	130	107
	*Cue*-*only*	*1068*	*175*	*1116*	*195*	*47*	*58*
1000, 2400 ms	Completed	783	132	848	150	65	133
	*Cue*-*only*	*951*	*161*	*962*	*170*	*11*	*103*
Error (%)							
Experiment 1							
300 ms	Completed	5.56	6.01	10.35	7.06	4.79	5.39
	*Cue*-*only*	*8.04*	*6.75*	*9.60*	*6.93*	*1.57*	*6.21*
1000, 2400 ms	Completed	8.21	6.89	11.12	7.94	2.91	5.02
	*Cue*-*only*	*8.65*	*8.14*	*10.10*	*7.68*	*1.45*	*6.42*
Experiment 2							
0 ms	Completed	4.01	4.52	7.37	5.31	3.36	3.97
	*Cue*-*only*	*4.93*	*5.52*	*8.83*	*8.40*	*3.90*	*6.99*
1000, 2400 ms	Completed	4.41	3.94	7.94	6.26	3.54	5.90
	*Cue*-*only*	*5.63*	*5.99*	*6.29*	*4.93*	*0.66*	*6.04*
Experiment 3							
0 ms	Completed	4.75	4.61	9.95	8.36	5.20	7.12
	*Cue*-*only*	*5.00*	*5.34*	*5.36*	*5.10*	*0.36*	*3.76*
1000, 2400 ms	Completed	6.24	8.45	12.06	11.42	5.83	10.38
	*Cue*-*only*	*7.08*	*7.73*	*8.02*	*6.96*	*0.94*	*5.56*

Data from trials preceded by cue-only trials are shown in italics

Switch costs were calculated prior to rounding. These data are controlled for cue-cue interval as described in the text, and are relevant to Analyses 1 and 2 in each experiment

**Table 2:** Means (M) and standard deviations (SD) of performance and switch cost scores (RT and % errors) on trials with a short current preparation interval (300 ms, Expt. 1; 0 ms, Expt. 2) in Experiments 1 and 2, according to preceding preparation interval and preceding trial completion

Experiment/preceding preparation interval	Preceding trial completion	Repeat		Switch		Switch cost	
		M	SD	M	SD	M	SD
RT (ms)							
Experiment 1							
1000 ms	Completed	839	106	876	113	38	66
	*Cue*-*only*	*854*	*123*	*950*	*120*	*96*	*89*
2400 ms	Completed	852	113	880	121	29	85
	*Cue*-*only*	*887*	*147*	*927*	*133*	*40*	*84*
Experiment 2							
1000 ms	Completed	1030	189	1090	192	60	89
	*Cue*-*only*	*1124*	*202*	*1241*	*238*	*117*	*117*
2400 ms	Completed	1058	192	1099	198	41	94
	*Cue*-*only*	*1159*	*219*	*1227*	*221*	*67*	*136*
Error (%)							
Experiment 1							
1000 ms	Completed	5.50	5.85	10.07	6.80	4.57	4.90
	*Cue*-*only*	*7.32*	*6.54*	*10.65*	*7.39*	*3.34*	*5.66*
2400 ms	Completed	5.87	6.02	10.25	6.71	4.38	6.11
	*Cue*-*only*	*8.04*	*6.75*	*9.60*	*6.93*	*1.57*	*6.21*
Experiment 2							
1000 ms	Completed	4.06	4.61	7.07	5.22	3.01	3.86
	*Cue*-*only*	*5.09*	*4.74*	*9.40*	*9.25*	*4.32*	*6.67*
2400 ms	Completed	3.99	4.06	6.02	5.66	2.03	4.31
	*Cue*-*only*	*4.93*	*5.52*	*8.83*	*8.40*	*3.90*	*6.99*

Data from trials preceded by cue-only trials are shown in italics

No control was imposed over cue-cue interval in these data. It is the switch cost scores (not the repeat and switch values themselves) that are relevant to Analysis 3 in Experiments 1 and 2

### Analysis 1: Switch costs at the short preparation interval (300 ms); CCI-controlled^1^

RT switch costs were significant following both completed trials (mean cost 44 ms, 95% CI [19, 68]), *t*(31) = 3.57, *p* = 0.001, *d*_*z*_ = 0.63, and cue-only trials (mean cost 40 ms [10, 70]), *t*(31) = 2.70, *p* = 0.011, *d*_*z*_ = 0.48. There was no significant difference between these costs (i.e., no significant interaction between preceding trial completion and transition), *F*(1,31) = 0.03, *p* = 0.86, $\eta_{G}^{2}$ < 0.001 (BF_10_ = 0.192). The main effect of transition was significant, with an overall cost for switching tasks, *F*(1,31) = 21.35, *p* < 0.001, $\eta_{G}^{2}$ = 0.03, as was the main effect of preceding trial completion, due to responses being slower following cue-only than completed trials overall, *F*(1,31) = 24.36, *p* < 0.001, $\eta_{G}^{2}$ = 0.06.

Analysis of the percentage error data showed that the cost of switching tasks following completed trials was significant (mean cost 4.80%, 95% CI [2.85, 6.74]), *t*(31) = 5.03, *p* < 0.001, *d*_*z*_ = 0.89, but following cue-only trials was not (mean cost 1.57% [− 0.67, 3.81]), *t*(31) = 1.43, *p *= 0.16, *d*_*z*_ = 0.25. Correspondingly, there was a significant interaction between preceding trial completion and transition, with a smaller switch cost following cue-only than following completed trials, *F*(1,31) = 5.75, *p* = 0.02, $\eta_{G}^{2}$ = 0.01. The main effect of transition was significant, *F*(1,31) = 16.72, *p* < 0.001, $\eta_{G}^{2}$ = 0.05, but that of preceding trial completion was not, *F*(1,31) = 2.11, *p *= 0.16, $\eta_{G}^{2}$ = 0.004.

### Analysis 2: Effect of current preparation interval on switch costs; CCI-controlled (see footnote 1)

The analysis of RT data including the longer current preparation intervals (combined over 1000 and 2400 ms) showed that there was no significant three-way interaction between preparation interval, preceding trial completion and transition, *F*(1,31) = 0.05, *p* = 0.83, $\eta_{G}^{2}$ < 0.001 (BF_10_ = 0.193), and therefore no differential RISC effect according to preceding trial completion. The two-way interaction between preparation interval and transition was not itself significant, so there was no overall RISC effect in these data, *F*(1,31) = 0.19, *p* = 0.67, $\eta_{G}^{2}$ < 0.001. The main effect of preparation interval was significant, *F*(1,31) = 10.84, *p* = 0.002, $\eta_{G}^{2}$ = 0.03, and interacted significantly with preceding trial completion, *F*(1,31) = 32.38, *p* < 0.001, $\eta_{G}^{2}$ = 0.04. The latency of responses following cue-only trials reduced with increasing preparation interval on average by 91 ms, 95% CI [57, 124], *t*(31) = 5.44, *p* < 0.001, *d*_*z*_ = 0.96, but there was no such reduction following completed trials, − 1 ms [− 31, 30], t(31) = − 0.04, *p* = 0.97, *d*_*z*_ = − 0.01. Overall, there was a significant switch cost (main effect of transition), *F*(1,31) = 37.50, *p* < 0.001, $\eta_{G}^{2}$ = 0.03. The main effect of preceding trial completion was not significant, *F*(1,31) = 2.98, *p* = 0.09, $\eta_{G}^{2}$ = 0.004, and there was not a significant interaction between transition and preceding trial completion, *F*(1,31) < 0.01, *p* = 0.95, $\eta_{G}^{2}$ < 0.001.

Just as with the RT data, the RISC effect did not differ significantly according to preceding trial completion in the percentage error data (three-way interaction), *F*(1,31) = 0.85, *p* = 0.36, $\eta_{G}^{2}$ = 0.001 (BF_10_ = 0.279), and again no overall RISC effect (two-way interaction) was observed, *F*(1,31) = 0.92, *p* = 0.34, $\eta_{G}^{2}$ = 0.001. Preparation interval was not significant as a main effect, *F*(1,31) = 2.71, *p* = 0.11, $\eta_{G}^{2}$ = 0.006, and did not interact with preceding trial completion, *F*(1,31) = 1.46, *p* = 0.24, $\eta_{G}^{2}$ = 0.002. The error switch cost was significant overall, *F*(1,31) = 26.17, *p* < 0.001, $\eta_{G}^{2}$ = 0.03. There was no overall effect of preceding trial completion, *F*(1,31) = 0.22, *p* = 0.64, $\eta_{G}^{2}$ < 0.001, but the interaction between transition and preceding trial completion was significant, *F*(1,31) = 5.05, *p* = 0.03, $\eta_{G}^{2}$ = 0.007. Error switch costs following completed trials, 3.85%, 95% CI [2.31, 5.39], *t*(31) = 5.09, *p* < 0.001, *d*_*z*_ = 0.90, were on average larger than those following cue-only trials, 1.51% [0.04, 2.98], *t*(31) = 2.10, *p* = 0.044, *d*_*z*_ = 0.37.

### Analysis 3: Effect of preceding preparation interval on switch costs at the short preparation interval (300 ms); non-controlled (see footnote 1)

Analysis of the RT switch cost data revealed a significant main effect of preceding preparation interval, *F*(1,31) = 10.23, *p* = 0.003, $\eta_{G}^{2}$ = 0.04, a larger interval being followed by a smaller switch cost. This fits with the hypothesis that increasing cue-cue interval would tend to decrease switch costs. The effect of preceding trial completion was significant, switch cost being larger overall following cue-only trials than following completed trials, *F*(1,31) = 4.60, *p* = 0.04, $\eta_{G}^{2}$ = 0.04. This result therefore replicates the pattern seen in previous studies which also did not control cue-cue interval. The interaction between preceding preparation interval and preceding trial completion was not significant, *F*(1,31) = 3.42, *p* = 0.07, $\eta_{G}^{2}$ = 0.02.

Analysis of the percentage error data revealed neither a significant reduction in switch cost as the preceding preparation interval increased, *F*(1,31) = 0.91, *p* = 0.35, $\eta_{G}^{2}$  = 0.008, nor a significant interaction of preceding preparation interval with preceding trial completion, *F*(1,31) = 0.78, *p* = 0.38, $\eta_{G}^{2}$ = 0.005. The main effect of preceding trial completion was significant, with switch costs following cue-only trials being larger than those following completed trials, *F*(1,31) = 4.61, *p* = 0.04, $\eta_{G}^{2}$ = 0.03.

## Discussion

The main result from Experiment 1 is that when cue-cue interval was controlled, RT switch costs at the short preparation interval were approximately equal following cue-only and completed trials. When cue-cue interval was not controlled, in contrast, switch costs were larger following cue-only than completed trials, as in previous studies (Lenartowicz et al., 2011,2; Swainson et al., 2017). Further, increasing preparation interval on the preceding trial significantly decreased switch costs on the current trial. Together, these results indicate that unbalanced cue-cue intervals probably accounted at least partly for the surprisingly large switch costs following cue-only trials seen in previous studies.

In terms of the error data, the switch cost was significantly larger following completed than cue-only trials, when controlling cue-cue interval, providing possible support for the hypothesis that performance *does* contribute something to the size of the subsequent switch cost that preparation alone does not. However, we note that this effect was not to be replicated in Experiment 2 (below).

We analysed RISC effects (with data controlled for cue-cue interval) to test whether switch costs following preparation versus performance would be differentially vulnerable to preparation on the current trial. We hypothesised that the cost following preparation might be more easily (and therefore more rapidly) overcome during preparation on the current trial than the cost following performance would. We saw no evidence of this. There was no RISC effect at all evident in the RT data; in the error data, it was the switch cost following completed trials that showed the larger RISC effect. This may have been simply due to this cost being higher at the 300 ms preparation interval, and therefore having further to fall towards zero than was the case for the analogous switch cost following cue-only trials.

With respect to what these results can say about the relative effects of preparation versus performance on the size of subsequent switch costs, it is important to note that we did not *match* cue-cue intervals between preceding trial completion conditions in this design. Rather, we replaced a bias in one direction in the previous studies (larger interval following completed trials) with a somewhat smaller bias in the other direction here.^3^ So should we now suspect that the switch cost following cue-only trials is now relatively *under*-estimated and that it is in fact at least as large as that driven by performance? Before drawing such a conclusion, we need to consider that we may have been unable to observe the full switch cost in this design. The shortest preparation interval was 300 ms (as it had been in Expt. 2 of Swainson et al., 2017; in Lenartowicz et al., 2011, Expt. 2, it was 350 ms). This may have been long enough to allow a substantial part of the switch cost driven by the preceding trial to be “prepared away” before target onset, as is seen in the RISC effect. Any part of the switch cost eliminated prior to target onset would not be captured within the RT. Hence, it could be that some aspect of the total switch cost might (consistent with two-stage task-switching theory) be driven only by performance, but that even the short preparation interval was too long to capture it in this experiment. This possibility was disallowed in Experiment 2, where the short preparation interval was 0 ms.

## Experiment 2

This experiment and its aims were very similar to those of Experiment 1. The main difference was that all trials with a 300 ms preparation interval (these were all completed trials) were replaced with trials on which the preparation interval was 0 ms—i.e., the cue and target appeared simultaneously. Again, we aimed to assess the following: the presence and relative size of switch costs following completed versus cue-only trials, when measured with a short (zero) preparation interval; whether there would be a difference in how rapidly those costs could be overcome with increasing preparation time on the current trial; and whether increasing cue-cue intervals diminish the switch cost.

## Method

### Participants

Thirty-nine participants (31 female, 8 male) were tested in total, in return for course credit or monetary reimbursement. The age range was 17–25 years (median 19 years).

### Materials

These were the same as for Experiment 1 except that the font size for cues was reduced to 28 point to improve the visual balance between cues and targets.

### Procedure

This was based closely on that for Experiment 1, with a few key differences. The 300 ms preparation intervals were all replaced with 0 ms intervals. On these trials, the cue and target were displayed simultaneously with the cue word overlaid upon the target (see Fig. 1). The durations of the cue and target stimuli on all trials were increased to 400 ms to prevent trials with no preparation from being too difficult. The duration of the blank screen that followed cues was correspondingly reduced to 600 ms and 2000 ms, so that the overall preparation intervals remained as 1000 ms and 2400 ms. Incorrect responses were signalled by the screen turning pink for 500 ms and then black for 2000 ms. As in Experiment 1, although we had intended there to be no response-cue interval here, a short, variable delay was found to have occurred, while the software processed a response and began the next trial. This delay was below 80 ms on 99% trials, with a mean of 37 ms prior to trials with preparation and 46 ms prior to trials with no preparation. It did not differ according to whether it preceded switch or repeat trials.

### Data processing

We tested participants until we had data from 36 participants following exclusion and replacement of participants (see below).^4^ This sample size allowed us to test for effects with at least a “medium” effect size (Cohen’s *d* = 0.5) with 80% power, whilst allowing full counterbalancing of colour and shape feature-response mappings in the analysed dataset. Trials with RTs below 200 ms or above 3000 ms were excluded from analysis. (N.B., the original upper limit of 2000 ms would have led to exclusion of a large number of participants, because responses were slower overall than in Experiment 1, probably due to the simultaneous presentation of cues and targets on some trials.) Trials following an incorrect response and the first trial of every block were also excluded. Trials with an incorrect response were excluded from the analysis of RTs. No participants had to be excluded for achieving less than 70% accuracy overall on completed trials in experimental blocks. Three participants were excluded because more than 10% trials were removed from the analysis for being too fast or too slow; these were replaced with new participants using the same feature-response mappings. A mean of 2% trials (range 0–9%) across participants were excluded from the final dataset for being too fast or too slow. As in Experiment 1, no limit was set for the number of trials per participant available for each analysis condition, and since the data processing pathway was preregistered, none was imposed at the analysis stage. A post hoc analysis of trial numbers showed that at least 20 trials were included per analysed condition for each participant except for in a single case (one condition, for one participant, relevant to Analysis 3 only), where only 16 trials were available.

Cue-cue interval was controlled for as in Experiment 1. Across participants, a mean of 15% trials (range 1–38%) were discarded to ensure that preceding completed trials (preparation interval 1000 ms) had RTs no longer than 1400 ms.

### Design and analysis

These exactly match the design and analysis of Experiment 1.^5^

## Results

Data relevant to Analyses 1 and 2 are shown in Table 1 and Fig. 2. Data relevant for Analysis 3 are shown in Table 2.

### Analysis 1: Switch costs at the short preparation interval (0 ms); CCI-controlled (see footnote 1)

RT switch costs were significant following both completed trials (mean cost 53 ms, 95% CI [20, 85]), *t*(35) = 3.27, *p* = 0.002, *d*_*z*_ = 0.55 and cue-only trials (mean cost 67 ms [21, 113]), *t*(35) = 2.97, *p* = 0.005, *d*_*z*_ = 0.50. Although the switch cost following cue-only trials was numerically larger than that following completed trials, there was not a significant difference, *F*(1,35) = 0.50, *p* = 0.48, $\eta_{G}^{2}$ < 0.001 (BF_10_ = 0.226). Overall, responses on switch trials were slower than on repeat trials, *F*(1,35) = 13.04, *p* < 0.001, $\eta_{G}^{2}$ = 0.02, and responses were slower when the preceding trial was cue-only than when it was completed, *F*(1,35) = 45.59, *p* < 0.001, $\eta_{G}^{2}$ = 0.12.

Percentage error switch costs were significant following both completed trials (mean cost 3.36%, 95% CI [2.02, 4.70]), *t*(35) = 5.08, *p *< 0.001, *d*_*z*_ = 0.85, and cue-only trials (mean cost 3.90% [1.54, 6.27]), *t*(35) = 3.35, *p* = 0.002, *d*_*z*_ = 0.56. Although the switch cost following cue-only trials was numerically the larger of the two, there was no significant difference, *F*(1,35) = 0.19, *p *= 0.67, $\eta_{G}^{2}$ < 0.001 (BF_10_ = 0.196). Overall, there was a higher percentage of errors on switch trials than on repeat trials, *F*(1,35) = 25.95, *p *< 0.001, $\eta_{G}^{2}$ = 0.08. There was no overall effect of preceding trial completion, *F*(1,35) = 3.04, *p* = 0.09, $\eta_{G}^{2}$ = 0.01.

### Analysis 2: Effect of current preparation interval on switch costs; CCI-controlled (see footnote 1)

Numerically, the RISC effect in RTs was slightly larger following cue-only trials than following completed trials; however, this difference (i.e., the three-way interaction) was not statistically significant, *F*(1,35) = 1.16, *p* = 0.29, $\eta_{G}^{2}$ < 0.001 (BF_10_ = 0.306). The RISC effect was significant overall in this experiment, *F*(1,35) = 6.29, *p* = 0.02, $\eta_{G}^{2}$ = 0.004. The main effects of preceding trial completion, *F*(1,35) = 28.87, *p* < 0.001, $\eta_{G}^{2}$ = 0.04, and preparation interval, *F*(1,35) = 180.96, *p* < 0.001, $\eta_{G}^{2}$ = 0.29, were both significant: RTs were faster following completed than cue-only trials and on trials with a preparation interval (1000,2400 ms combined) than on trials without (0 ms). These two factors interacted significantly, *F*(1,35) = 34.67, *p* < 0.001, $\eta_{G}^{2}$ = 0.05, showing that RTs reduced more with increasing preparation interval following cue-only than following completed trials. The switch cost was significant overall, *F*(1,35) = 14.30, *p* < 0.001, $\eta_{G}^{2}$ = 0.01, and was not significantly affected by preceding trial completion, *F*(1,35) < 0.01, *p* = 0.96, $\eta_{G}^{2}$ < 0.001.

In the error data, although numerically it appeared that the RISC effect following cue-only trials might be steeper than that following completed trials, the three-way interaction effect was not significant, *F*(1,35) = 2.71, *p* = 0.11, $\eta_{G}^{2}$ = 0.006 (BF_10_ = 0.609). Only the main effect of transition (indicating an overall switch cost) was significant in this analysis, *F*(1,35) = 34.07, *p* < 0.001, $\eta_{G}^{2}$ = 0.06. All other effects were non-significant: main effects of preparation interval, *F*(1,35) = 0.21, *p* = 0.65, $\eta_{G}^{2}$ < 0.001, and preceding trial completion, *F*(1,35) = 1.57, *p* = 0.22, $\eta_{G}^{2}$ = 0.002; and the two-way interactions between preparation interval and preceding trial completion, *F*(1,35) = 1.70, *p* = 0.20, $\eta_{G}^{2}$ = 0.004, between preparation interval and transition, *F*(1,35) = 2.41, *p* = 0.13, $\eta_{G}^{2}$ = 0.005, and between preceding trial completion and transition, *F*(1,35) = 1.82, *p* = 0.19, $\eta_{G}^{2}$ = 0.003.

### Analysis 3: Effect of preceding preparation interval on switch costs at the short preparation interval (0 ms); non-controlled (see footnote 1)

As in Experiment 1, analysis of the RT data (see Table 2) showed that a larger preparation interval on the preceding trial led to smaller switch costs on the current trial, *F*(1,35) = 5.83, *p* = 0.02, $\eta_{G}^{2}$ = 0.02. This effect did not interact significantly with preceding trial completion, *F*(1,35) = 1.29, *p* = 0.26, $\eta_{G}^{2}$ = 0.005. Switch costs following cue-only trials were larger overall than those following completed trials in this analysis, *F*(1,35) = 6.46, *p* = 0.02, $\eta_{G}^{2}$ = 0.04, mirroring the analogous result in Experiment 1.

No effects were significant in the analysis of percentage error data: main effect of preceding preparation interval, *F*(1,35) = 0.97, *p* = 0.33, $\eta_{G}^{2}$ = 0.004; main effect of preceding trial completion, *F*(1,35) = 2.34, *p* = 0.13, $\eta_{G}^{2}$ = 0.02; interaction, *F*(1,35) = 0.13, *p* = 0.72, $\eta_{G}^{2}$ < 0.001.

## Discussion

The outcome of Experiment 2 was similar to that of Experiment 1. With control over cue-cue interval, switch costs on trials with no preparation interval were roughly equivalent in size between preceding trial completion conditions (numerically slightly larger following cue-only than completed trials) and were significant following both types of trial. Therefore, we have no reason to think that part of the performance-driven switch cost had been rendered unobservable by being prepared away prior to target onset in earlier experiments. More importantly, we again did not find any evidence that performance adds anything to the size of the subsequent switch cost at short/zero (current) preparation intervals, over and above that contributed by preparation alone.

As in Experiment 1, when we reanalysed the data without controlling cue-cue interval, we found that a significantly longer preparation interval on the preceding trial (and therefore a longer cue-cue interval) led to significantly smaller switch costs on the current trial and the relatively large switch cost following cue-only trials re-emerged. Hence, this result again supports the idea that unbalanced cue-cue intervals had contributed to the large switch costs following cue-only trials in previous studies (Lenartowicz et al., 2011; Swainson et al., 2017).

Unlike in Experiment 1, the RISC effect (analysed in data controlled for cue-cue interval) was significant overall here (in RTs), possibly because the 0 ms preparation interval in this experiment provided a greater contrast with the long (1000 ms and 2400 ms combined) preparation interval condition. Numerically, the pattern of RISC effects across preceding trial completion conditions (i.e., a bigger RISC effect following cue-only than completed trials) appeared consistent with our hypothesis that switch costs driven only by preparation on the preceding trial might be more easily overcome during preparation on the current trial than those driven by performance would. However, the interaction was not significant in either the RT or the error data. Future studies (perhaps with greater power to measure switch costs across a range of preparation intervals) may be able to offer a more conclusive answer to this question.

To return to the question of the relative magnitude of switch costs at short/zero preparation intervals, can we now conclude that these are at least as large following cue-only trials as following completed trials? We wished to rule out a further confound before drawing this conclusion. In Experiment 3, we introduced a new design to control for the differential expectancies of event types following cue-only and completed trials that had been present in previous experiments.

## Experiment 3

So far, in the studies of subsequent switch costs using cue-only or no-go trials, those trials have been relatively rare. (For example, 30% of long preparation trials were cue-only in Experiments 1 and 2 here and also in Swainson et al., 2017; 25% of all trials were cue-only in Lenartowicz et al. 2011, Expt. 2; 25% of all trials were no-go in Schuch and Koch, 2003). There is a good reason for making these trials rare: participants may well be less likely to prepare a task in advance of target presentation if there is a high likelihood that a target will never appear on that trial. If there is no preparation, its effects on the subsequent trial cannot be tested. However, the rarity of cue-only/no-go trials, together with the restriction that these trials never occur consecutively, causes an imbalance in the relative frequency of subsequent events, as follows. Following a completed trial, there is a 100% likelihood that the next stimulus event will be the onset of a task-cue, with a 50% likelihood of that cue signalling a task-switch. Assuming that participants are sufficiently sensitive to these probabilities, we might say that following a completed trial they will be “50% ready” to switch tasks. Following a cue-only trial, in contrast, participants will tend to expect a target to be presented next, rather than the next trial’s cue. This is because cue-only trials are identical to the preparation stage of completed trials and they occur much less frequently than completed trials do. Following a long preparation interval, there will be only a 30% chance that the next event will be the onset of a task-cue (versus 70% chance of it being a target), half of these cues (15% of the total) signalling a task-switch. We might say therefore that participants are only “15% ready” to switch tasks following a cue-only trial. This difference in switch-readiness (50% vs. 15%) following completed versus cue-only trials might mean that any cost of task-switching is increased in the rarer condition, because the participant has to first make a larger adjustment to the idea that there is a need to switch tasks before actually doing so. This adjustment could feasibly contribute to the switch cost measured following cue-only trials.

To equate the frequencies of cue and target events across a block of trials, we made substantial changes to the usual structure of trials adopted in cued task-switching designs. Instead of construing a standard trial as a sequence of cue, target and response events we instead construe a trial here as one of the following: (i) a simultaneous cue and target (plus a response); (ii) a cue only (plus preparation time); (iii) a target only (plus a response). We dropped the requirement for cue-only trials not to occur consecutively and instead presented the three “trial” types with equal likelihood following every type of trial. During data analysis, we identified sequences of these “trials” which corresponded to the same sequences of trial events constituting the trial sequences analysed in Experiments 1 and 2.

The inclusion of target-only trials, where the task from the preceding trial would necessarily repeat, had another beneficial effect. Following response-selection on any cue-target or target-only trial there was now a reason to maintain preparation of that trial’s task up until the presentation of the next stimulus event (in case the next trial was a target-only trial). Cue-only trials have always required the prepared task to be maintained in this way, but it has not been the case following completed trials (in Expts. 1 or 2 here, or in the previous cue-only studies by Lenartowicz et al., 2011 or Swainson et al., 2017). Hence, this is another way in which preceding trial completion conditions are better matched in this experiment than previously.^6^

## Methods

### Participants

Forty-two participants (36 female) were tested in total, in return for course credit or monetary reimbursement. The age range was 17–32 years (median 20 years).

### Materials

These were the same as for Experiments 1 and 2, except for the way that task-cues were presented. Cue-words (Arial Black font, size 20, presented in white) were displayed within a grey rectangle for visual clarity. When cue and target were simultaneous, the coloured-shape target was shown as if behind the grey rectangle (see Fig. 1).

### Procedure

Participants were tested across two testing sessions (with no restriction on time between sessions), each approximately 70–90 min long, in order to collect sufficient trials within key conditions. The procedure was identical across both sessions, including practice. Four participants did not complete all of both sessions due to time constraints (they completed at least 80% of the experimental trials), but their data were included as long as they were able to contribute sufficient trials (see below). Participants first practised the colour and shape tasks individually for 30 trials each (including all three trial-types—cue-target, cue-only and target-only). Then both tasks were intermixed, first for a 30-trial practice block and then a longer 102-trial practice block.^7^ There then followed 32 experimental blocks, each composed of 76 trials. After every block, participants were informed of the number of errors they had made and their mean RT during that block and participants started the next block when they were ready. The after-block feedback was introduced in Experiment 3 in an attempt to motivate participants to perform well, and thereby to avoid having to exclude participants unnecessarily because of poor performance. As in Experiments 1 and 2, participants were encouraged to take a break after every block and to take longer breaks after every eight blocks.

Importantly, what constituted a trial in this experiment was very different from Experiments 1 and 2 and from standard task-switching experiments (see Fig. 1). On each trial, the trial-type was selected at random such that there was a 1/3 chance of each of the following trial types being selected: cue-target (CT); cue-only (CO); target-only (TO). On cue-target trials, the task-cue was shown superimposed upon a coloured-shape target for (a maximum of) 800 ms, followed by a blank screen until a response was registered. Cue-only trials consisted of a task-cue displayed for 800 ms, followed by a blank screen for either 200 or 1600 ms (with a 50% chance of each duration being selected), producing preparation intervals of 1000 and 2400 ms as in Experiments 1 and 2. Target-only trials consisted of only a coloured-shape target stimulus being presented for (a maximum of) 800 ms, followed by a blank screen until a response was registered. Participants were instructed to continue to use a cue to determine which task to use until another cue was shown, and the first trial in a block was never a target-only trial. On both cue-target and target-only trials, the execution of a response initiated either error feedback or the onset of the next trial’s display. As in the previous two experiments, although we had intended there to be no post-response interval, a short, variable delay (below 50 ms on 99% trials) was found to have occurred, while the software processed the preceding response and began the current trial. This delay was around 6 ms longer when cue and target were shown simultaneously on the current trial than when either a cue or target were shown alone (mean 33 ms vs. mean 27 and 28 ms, respectively), but importantly it did not differ between switch and repeat trials.

### Data processing (see footnote 8)

**Fig. 3:**
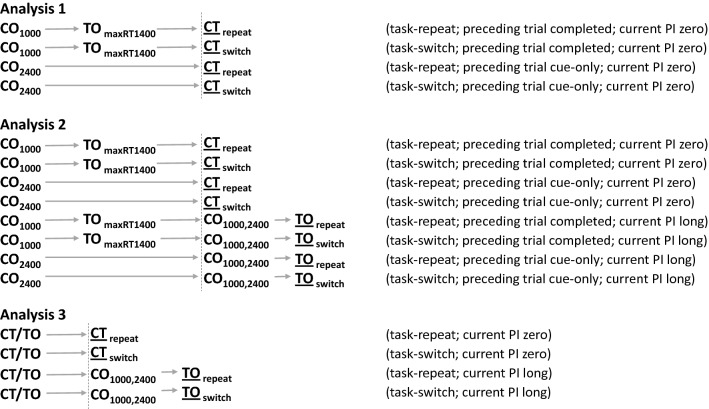
Trial sequences entered into Analyses 1–3 in Expt. 3. CT = cue-target; CO = cue-only; TO = target-only; PI = preparation interval. (N.B. CT trials and CO → TO trial sequences are both classified as “completed trials” in this experiment.) Spacing of items roughly represents the relative timings across trials. Trials shown to the left of the vertical dashed line combine to correspond to the “preceding trial” of Analyses 1 and 2, and those to the right combine to correspond to the “current trial” of those analyses. The underscored trial indicates that from which performance data were obtained for each analysed trial-sequence. N.B. Data in Analysis 3 were converted to switch cost scores before being statistically analysed

RTs below 200 ms or above 3000 ms were not analysed. Trials with an incorrect response were excluded from being the preceding trial of any analysed trial sequence (see Fig. 3), and from being the current trial in RT analyses. All trials contributing to the preceding and current trials had to fall entirely within the current block. Two participants who scored less than 70% correct overall on trials requiring a response (cue-target, target-only) in experimental blocks were excluded. No further participants had to be excluded on the basis of more than 10% of their trials being too fast or too slow. Since the specific trial sequences required for analysis were rare and occurred probabilistically, we wished to ensure that data were not included in analyses where they were averaged from only relatively few instances of that sequence type. Therefore, we also excluded participants from all analyses if they were able to contribute fewer than 20 of any of the trial sequences required for analysis (as shown in Fig. 3), following trial exclusions as described above. Four further participants were excluded for this reason. All excluded participants were replaced with new participants using the same feature-response mappings. A mean of 2% trials (range 0–7%) across participants were excluded from the final dataset for being too fast or too slow.

Cue-cue interval was controlled for as in Experiments 1 and 2. Across participants, a mean of 13% trials (range 0–29%) were discarded to ensure that preceding RTs for completed tasks were no longer than 1400 ms.

### Design and analysis^8^

Analyses 1 and 2 were equivalent to those in Experiments 1 and 2. Analysis 3 was different, however: in this experiment, this analysis looked specifically for evidence of pre-target task preparation. Figure 3 shows the sequences of trials used in these analyses.

Analyses 1 and 2 required that we defined sequences of trials to correspond to the “cue-only” and “completed” trial-types used in the previous experiments. Cue-only (CO) trials were already present in the new design. Completed trials were defined as corresponding to either cue-target (CT) trials or sequences of cue-only → target-only (CO → TO) trials. We controlled for cue-cue interval with an analogous procedure to that used in Experiments 1 and 2. Specifically, where the preceding trial-type was “completed”, the preparation interval on the CO trial of the CO → TO sequence was 1000 ms and the TO trial had a maximum RT of 1400 ms. Where the preceding trial-type was “cue-only”, its preparation interval was 2400 ms. (See Fig. 3, top and middle, for the relevant trial sequences).

Analysis 1 examined the switch cost on trials with no preparation interval (i.e., on cue-target trials) according to whether the preceding trial’s task had been performed or only prepared. The existence of the switch cost was tested for using *t* tests (repeat versus switch) within each preceding-trial-completion condition. These costs were compared with each other via a repeated-measures ANOVA with factors transition (repeat, switch) and preceding trial completion (completed, cue-only).

Analysis 2 examined the effect of the current preparation interval on switch costs. Preparation time for the current task was collapsed across the 1000 ms and 2400 ms intervals. These data were entered into a repeated-measures ANOVA with the factors transition^9^ (repeat, switch), preceding trial completion (completed, cue-only) and current trial preparation (0 ms, [1000 or 2400 ms, combined]).

Analysis 3 tested specifically for evidence (in the form of a RISC effect) that participants were preparing a specific task during the preparation interval. This analysis was included because the probability of having to implement a task cued on a cue-only trial was much lower in this paradigm than in the previous two experiments: only one-third of cue-only trials in the current paradigm would be followed by a target-only trial requiring that cued task to be implemented. The RISC effect (a reduced switch cost when preparation time was available on the current trial) should provide evidence that such preparation was taking place. Because we wanted to be able to use the results of this analysis to infer whether participants would have prepared tasks *on cue*-*only trials*, this analysis looked specifically at the RISC effect under exactly the same conditions that applied to cue-only trials, i.e., on trials following (any type of) completed trials. No control was imposed over cue-cue interval. The relevant trial sequences for this analysis are shown in Fig. 3 (bottom). A one-tailed *t* test examined whether the RISC effect ([switch cost on trials with no preparation]—[switch cost with either 1000 or 2400 ms preparation]) was significantly greater than 0 ms.

## Results

### Analysis 1: Switch costs at the short preparation interval (0 ms); CCI-controlled (see footnote 1)

RT switch costs were significant following both completed trials (mean cost 130 ms, 95% CI [94, 166]), *t*(35) = 7.28, *p* < 0.001, *d*_*z*_ = 1.21, and cue-only trials (mean cost 47 ms, [28, 67]), *t*(35) = 4.88, *p* < 0.001, *d*_*z*_ = 0.81. The repeated-measures ANOVA showed that the cost following completed trials was significantly larger than that following cue-only trials, *F*(1,35) = 23.97, *p* < 0.001, $\eta_{G}^{2}$ = 0.02. The overall switch cost was significant, *F*(1,35) = 58.12, *p *< 0.001, $\eta_{G}^{2}$ = 0.07; there was also a significant overall cost of the preceding trial being cue-only rather than completed *F*(1,35) = 151.23, *p *< 0.001, $\eta_{G}^{2}$ = 0.28.

In terms of percentage errors, while switch costs were significant following completed trials (mean cost 5.20%, 95% CI [2.79, 7.61]), *t*(35) = 4.39, *p *< 0.001, *d*_*z*_ = 0.73, they were not significant following cue-only trials (mean cost 0.36% [− 0.91, 1.63]), t(35) = 0.58, *p* = 0.57, *d*_*z*_ = 0.10. Correspondingly, the repeated-measured ANOVA showed a significant interaction between preceding trial completion and transition, *F*(1,35) = 18.60, *p* < 0.001, $\eta_{G}^{2}$ = 0.04. Overall, switch trials produced a higher percentage of errors than did repeat trials, *F*(1,35) = 13.23, *p* < 0.001, $\eta_{G}^{2}$  = 0.05, and there was a higher percentage of errors following completed trials than following cue-only trials, *F*(1,35) = 7.80, *p* = 0.008, $\eta_{G}^{2}$  = 0.03.

### Analysis 2: Effect of current preparation interval on switch costs; CCI-controlled (see footnote 1)

There was not a significantly different RISC effect in the RT data between preceding trial completion conditions (three-way interaction), *F*(1,35) = 0.74, *p* = 0.40, $\eta_{G}^{2}$ < 0.001 (BF_10_ = 0.252). Every other effect tested in this ANOVA, however, was significant. Thus, there was an overall switch cost, *F*(1,35) = 50.64, *p* < 0.001, $\eta_{G}^{2}$ = 0.04, that was significantly modified by preceding trial completion, *F*(1,35) = 15.96, *p* < 0.001, $\eta_{G}^{2}$ = 0.01, due to the switch cost (collapsed across preparation interval) being larger following completed trials (mean 97 ms) than following cue-only trials (29 ms). RTs were significantly faster overall following completed trials than following cue-only trials, *F*(1,35) = 127.88, *p* < 0.001, $\eta_{G}^{2}$ = 0.23. Increasing preparation time benefitted response speed, *F*(1,35) = 60.50, *p* < 0.001, $\eta_{G}^{2}$ = 0.10, and more so following completed trials than following cue-only trials, *F*(1,35) = 10.74, *p* = 0.002, $\eta_{G}^{2}$ = 0.009. Finally, the RISC effect itself was significant overall, *F*(1,35) = 8.52, *p* = 0.006, $\eta_{G}^{2}$ = 0.006: RISC effect overall, 51 ms. (Analysis 3 below describes the separate analysis of the RISC effect in RT data following completed trials only.)

In the error data, the three-way interaction was not significant, *F*(1,35) < 0.01, *p* = 0.98, $\eta_{G}^{2}$ < 0.001 (BF_10_ = 0.179). Hence, as with the RT data, there was no statistical evidence for the idea that switch costs following cue-only trials are more easily overcome than those following completed trials. Overall, there was a significant cost of switching tasks, *F*(1,35) = 20.00, *p* < 0.001, $\eta_{G}^{2}$ = 0.04. This effect interacted significantly with that of preceding trial completion, *F*(1,35) = 24.57, *p* < 0.001, $\eta_{G}^{2}$ = 0.03, such that the switch cost was larger following completed trials (mean 5.51%) than cue-only trials (0.65%). There were fewer errors overall following cue-only than completed trials, *F*(1,35) = 4.49, *p* = 0.04, $\eta_{G}^{2}$ = 0.02. The overall effect of preparation interval was, unusually, that errors were more common with a long than with a short preparation interval, *F*(1,35) = 12.13, *p* = 0.001, $\eta_{G}^{2}$ = 0.02. The effect of preparation interval did not interact with preceding trial completion, *F*(1,35) = 0.31, *p* = 0.58, $\eta_{G}^{2}$ < 0.001, or with transition, *F*(1,35) = 0.20, *p* = 0.65, $\eta_{G}^{2}$ < 0.001; thus, there was not a significant overall RISC effect in the error data (average RISC − 0.60%). (Analysis 3 below describes the separate analysis of the RISC effect in error data following completed trials only.)

N.B. In the above analyses, we collapsed data over the two “long” preparation intervals (1000 ms and 2400 ms) to achieve sufficient trial numbers per condition per participant (see data processing). Since switch costs were not identical across these intervals, we report them here for information only, as indicated in the preregistration document. Mean RT switch costs were as follows: following completed trials at the 1000 ms preparation interval (PI), 69 ms, 95% CI [9, 129] and at 2400 ms PI, 49 ms [− 26, 124]; following cue-only trials at 1000 ms PI, − 7 ms, [− 52, 37] and at 2400 ms PI, 30 ms, [− 11, 72]. Mean % error switch costs were as follows: following completed trials at 1000 ms PI, 7.77%, 95% CI [1.35, 14.18] and at 2400 ms PI, 0.23% [− 8.77, 9.23]; following cue-only trials at 1000 ms PI, − 0.40%, [− 3.15, 2.34] and at 2400 ms PI, 2.52%, [0.06, 4.98].

### Analysis 3: RISC effect following completed trials only; non-controlled (see footnote 1)

The data for this analysis are shown in Table 3 (switch cost column). The average RT RISC effect in this analysis was a substantial 69 ms, 95% CI [46, 92] and the one-tailed test for a positive RISC effect was significant, *t*(35) = 6.14, *p* < 0.001, *d*_*z*_ = 1.02. Thus, we have reason to believe that participants did use the preparation interval, when it was present, to prepare for the upcoming task. However, there was a small negative RISC effect in the error data (see Table 3) of − 1.24% errors, 95% CI [− 3.03, 0.54]. No one-tailed t-test was run on these data since the effect was not in the predicted direction.

**Table 3:** Data for Experiment 3, Analysis 3: RISC effect following completed trials only

Preparation interval	Repeat		Switch		Switch cost	
	*M*	*SD*	*M*	*SD*	*M*	*SD*
RT (ms)						
0 ms	854	141	981	158	126	55
1000, 2400 ms	900	157	957	162	57	50
Errors (%)						
0 ms	3.61	2.90	10.09	5.90	6.48	4.22
1000, 2400 ms	7.41	5.06	15.13	7.28	7.73	4.69

See Fig. 3 for relevant trial sequences from which these data were obtained. Switch costs were calculated prior to rounding

## Discussion

In Experiment 3, there was once again a significant cost of switching tasks following preparation alone (cue-only trials). For the first time in this series of studies, however, the switch cost following performance was significantly larger than that following preparation alone, and this was the case in terms of both RT and errors. At the 0 ms preparation interval, the RT cost was more than twice as large following completed trials than following cue-only trials (130 ms vs 47 ms). The implications of this finding for models of task-switching are discussed below (general discussion).

The switch cost at the short/zero preparation interval following completed trials (130 ms) was far larger in this experiment than it had been in both Experiments 1 (44 ms) and 2 (53 ms). This may be because the likelihood of having to switch tasks to process any target stimulus was lower than it had been in Experiments 1 and 2: this is because targets could now immediately follow other targets, all such instances requiring task repetition. It has been shown that reducing the overall likelihood of switching tasks increases the measured switch cost, perhaps because participants are less likely to switch preemptively before the next cue is shown (Monsell and Mizon, 2006). There was no corresponding increase following cue-only trials: the switch cost here was 47 ms at the short/zero preparation interval compared with 40 ms in Experiment 1 and 67 ms in Experiment 2. Possibly, any effect of reduced switch:repeat ratio in terms of increasing the subsequent switch cost was counteracted by an opposite effect, decreasing the cost following cue-only trials specifically. That is, there was an increased likelihood in this experiment (relative to Experiments 1 and 2) of a cue presented alone being followed by another cue (rather than by a target), thereby increasing the relative likelihood of an immediate subsequent task-switch.

There was no evidence here to support the hypothesis that switch costs would be more rapidly overcome during preparation on the current trial following preparation versus performance of the preceding task. The RT RISC effect was in fact numerically larger following completed than cue-only trials (the opposite of the effect required to support that hypothesis) and there was neither an overall nor a differential RISC effect in the error data.

## General discussion

In this series of experiments, we aimed to compare the effects of preparing a task with those of completing a prepared task on the subsequent switch cost, while removing or otherwise controlling for a number of confounds that had been present in previous studies. It seemed plausible that those confounds could have made the switch cost seen previously following preparation unrealistically large relative to that seen following performance. Across the three experiments, we found that the relative size of switch costs shifted from being bigger following preparation to being bigger following performance, while a significant cost remained following both preparation and performance in all three experiments.

The relative size of switch costs subsequent to preparing versus performing a task is highly relevant to the question of the extent to which our behaviour is controlled by voluntary, endogenous factors on the one hand and performance-related or exogenous factors on the other. Two-stage models of task-switching (e.g. Meiran, 2000; Rogers and Monsell, 1995; Rubinstein, Meyer, and Evans, 2001) posit that volition is limited in terms of enabling us to overcome a switch cost, with some aspect of task performance being required to fully switch between tasks. Analogously, one might expect a similar separation in terms of establishing a subsequent switch cost, such that whereas preparation might produce some cost of switching on the next trial, it should not be able to produce the full cost unless accompanied by some aspect of performance such as response-selection (c.f. Lenartowicz et al., 2011; Schuch and Koch, 2003). However, the data thus far have not borne out this expectation. Studies where, on the preceding trial, preparation was followed by a no-go stimulus have tended to show completely absent switch costs on the current trial (thereby favouring models where persisting effects stem from performance rather than preparation, e.g., Allport and Wylie, 2000). Studies with cue-only trials, where preparation is abruptly followed by the next trial, have tended to show that switch costs are larger, not smaller, than those seen following performance (thereby favouring models where persisting effects stem from preparation rather than performance, e.g., Altmann and Gray, 2008).

In this set of studies, we consistently found (as have previous studies with cue-only trials) significant switch costs to be present following trials on which a task was prepared but not performed. Hence, these data add to the weight of evidence that performance-related processes such as response-selection are not a necessary precondition for subsequent switch costs to exist, at least at short/zero current preparation intervals. We concur with Lenartowicz, Yeung, and Cohen (2011) in concluding that preparation is sufficient to drive subsequent switch costs. This finding fits both with two-stage and preparation-based models inasmuch as it indicates that endogenous task-preparation has a significant impact on task-readiness, and it seems rather less consistent with models where processes occurring during performance are responsible for driving subsequent switch costs (e.g. Allport and Wylie, 2000).

However, we were initially unable to generate switch costs that were larger following performance than following preparation alone. That is the situation one might expect if, as the two-stage (as well as the largely performance-based) models suggest, performing a task results in a more profound shift in our relative readiness to perform that task again than preparation alone can produce. In Experiments 1 and 2, removing a substantial response-cue interval and controlling for cue-cue intervals resulted only in switch costs becoming equivalent between conditions. In Experiment 3, we also matched the likelihood of a task-switch immediately following preparation versus performance of a task and here the predicted pattern emerged. Switch costs were significantly larger following completed than following cue-only trials. These data are consistent with a mechanism whereby task-performance affects an aspect of task-readiness that remains unaffected by volition alone. These data also suggest that the patterns seen in previous experiments, where switch costs following cue-only trials were larger than those following completed trials, may well have been produced by unbalanced timing and predictability factors. Hence, we suggest these data show that two-stage task-switching models are consistent with research showing even large switch costs following cue-only trials.

It might seem as though our data from Experiment 3, showing increase of switch cost following trials that involve performance as well as preparation, must contradict models that place the weight of the switch cost at the stage of preparation, such as that of Altmann and Gray (2008; see also Schneider and Logan, 2005). But we do not think that is necessarily so. We do not claim to have eliminated all potential biases that might affect the relative sizes of switch costs, and it is possible therefore that we are now underestimating the size of the switch cost following preparation relative to that following performance. First, we were not able to match cue-cue interval (CCI) across preceding trial completion conditions in any of our experiments: in our CCI-controlled analyses this interval was always somewhat longer following cue-only trials than following completed trials. As our analyses show (and as Altmann and Gray’s model seems to predict), longer intervals between the cue on the preceding trial and the cue on the current trial tend to reduce the size of switch costs. Second, it is possible that participants might actually have prepared to a lesser extent, or less consistently across trials, on cue-only trials than they did on completed trials. Although we took steps to encourage advance preparation (including using only a short cue duration; Verbruggen, Liefooghe, Vandierendonck, and Demanet, 2007), participants might potentially have delayed part of the preparation process until after target onset rather than preparing fully in advance, on at least some cue-only trials. If this was the case, these participants would have on average prepared to a greater extent by the end of completed than by the end of cue-only trials (since, presumably, preparation of the cued task would have had to take place at some point for that task to be used on a completed trial). It is plausible, therefore, that the increased size of the switch cost following completed (versus cue-only) trials seen in Experiment 3 could be partly due to the subsequent effects of increased preparation, rather than only of additional performance, on completed trials. This issue is potentially important wherever a condition ostensibly measuring the effect of preparation, but not actually requiring it, is compared with a condition on which preparation is obligatory. For instance, Verbruggen, McAndrew, Weidemann, Stevens and McLaren (2016) showed that motor excitability and costs of switching from no-go to go responses were apparently driven by performance on the preceding trial rather than by what could have been very confidently predicted to happen on the current trial. Those authors point out that top–down control is effortful, and so will not always be undertaken. Indeed, one of the key explanations of the residual switch cost is that of de Jong (2000; also cited by Verbruggen et al., 2016), whereby occasional “failures-to-engage” during a preparation interval, possibly due simply to a lack of motivation, create what appears to be a fundamental restriction on the limits of endogenous control during task-switching. Altmann and Gray’s (2008) model uses the failure-to-engage idea to produce the residual switch cost, with the cost stemming from occasional lapses in pre-target cue identification on switch trials without there being any requirement for a performance-driven process to complete a task-switch. In our experiments, then, it is simply not clear yet whether performance necessarily produces a larger subsequent switch cost than preparation does (in line with two-stage switching theory) or whether there may be a single switching process that can occur during preparation, responsible for driving the full subsequent switch cost, but which is more likely to take place when a task has to be completed than when it does not.

We have focussed primarily upon the size of switch costs measured on trials with short preparation intervals, because this is where the unexpectedly large costs following cue-only trials have been seen before (Lenartowicz et al., 2011; Swainson et al., 2017). However, it might be argued that any additional subsequent effects of performance should only be evident at longer preparation intervals, since it is the “residual” switch cost (that remaining after substantial preparation time on the current trial) that, by definition, is only eliminated by performance (e.g. Rogers and Monsell, 1995). In practice, however, the residual cost measure is a difficult one to use, since it is not always easy to determine when sufficient preparation time has been provided to enable the residual cost to be reached (c.f. Swainson and Martin, 2013). In the current experiments, we chose to use the steepness of decline in switch cost with increasing preparation interval (i.e., the size of the RISC effect) as an alternative way of assessing whether preparation and performance generated different types of subsequent switch cost. We reasoned that a cost driven only by prior preparation might be rapidly overcome during preparation on the current trial, whereas one that was driven by prior performance might be resistant to the effects of current preparation alone. There was a hint in the data from Experiment 2 that such a pattern existed (a numerically steeper RISC effect following cue-only than completed trials), but it was not supported statistically. Further research in this direction might prove fruitful to determine whether the nature of the switch cost preceded only by task-preparation differs qualitatively from that preceded by performance of a prepared task.

## Conclusion

These experiments confirm that preparing but not performing a particular task is sufficient to produce a switch cost on the next trial. The relative size of the switch costs following preparation versus performance changed across the series of experiments, consistent with the hypothesis that unbalanced timings and event probabilities had contributed to the previous finding of surprisingly large switch costs following cue-only trials. The larger switch cost following performance than following preparation seen in the third experiment is consistent with two-stage models of task-switching that propose a special role for performance in task-control. However, we may now be overestimating the size of the switch cost following completed trials relative to that following cue-only trials. Different methods may be required to establish whether there are meaningful qualitative differences in the types of switch cost driven by preparation versus performance.

## Data Availability

Data for all three experiments are available on the Open Science Framework, https://osf.io/auvt6/.

## Footnotes

1 “CCI-controlled” means that the analysis controls for cue-cue interval between preceding-trial-completion conditions (N.B. it does not fully match CCI: seeM “ethods” section for details). “Non-controlled” means that no such control was applied.

2 N.B. This effect in Lenartowicz et al. (2011) was not tested for significance, but was of a similar relative size to that found to be significant in Swainson et al. (2017).

3 In this experiment, cue-cue interval following completed trials was around 1850 ms (1000 ms preparation interval plus approx. average RT 850 ms), whereas that following cue-only trials was 2400 ms; hence, the difference between conditions in cue-cue interval was approximately 550 ms on average in this experiment. The equivalent difference in Swainson et al. (2017, Expt 2) was 2000 ms in the opposite direction.

4 The data-processing procedure was preregistered on the Open Science Framework, https://osf.io/4zwb8, where this experiment is termed “Experiment 1”.

5 Only the intention to run the three-way ANOVA used in Analysis 2 was preregistered.

6 We thank Agnieszka Kowalczyk for pointing this out.

7 The length of this practice block had been intended to match the experimental blocks but was coded as 102 trials in error.

8 Data-processing procedure and analyses were preregistered on the Open Science Framework, https://osf.io/yqc9q/

9 For this analysis, the term “transition” refers to the relationship between the task relevant to the current trial on which performance is measured (always a CT or TO trial) and the previously cued task. This means that for sequences where the current task was prepared, i.e., [CT or TO] → CO → TO, transition is between the task on the current TO trial and the task on trial *n* – 2, CT or TO, and not the task on trial *n* − 1, which is effectively acting as the cue for the current trial.

## References

[CR1] Allport A, Styles EA, Hsieh S, Umilta C, Moscovitch M (1994). Shifting intentional set: Exploring the dynamic control of tasks. Attention and performance XV: Conscious and nonconscious information processing.

[CR2] Allport A, Wylie G, Monsell S, Driver J (2000). Task-switching, stimulus-response bindings, and negative priming. Control of cognitive processes: Attention and performance XVIII.

[CR3] Altmann EM, Gray WD (2008). An integrated model of cognitive control in task switching. Psychological Review.

[CR4] Astle DE, Jackson GM, Swainson R (2006). Dissociating neural indices of dynamic cognitive control in advance task-set preparation: An ERP study of task switching. Brain Research.

[CR5] Brass M, Liefooghe B, Braem S, De Houwer J (2017). Following new task instructions: Evidence for a dissociation between knowing and doing. Neuroscience and Biobehavioral Reviews.

[CR6] Brass M, von Cramon D (2002). The role of the frontal cortex in task preparation. Cerebral Cortex.

[CR7] Bugg JM, Scullin MK (2013). Controlling intentions: The surprising ease of stopping after going relative to stopping after never having gone. Psychological Science.

[CR8] de Jong R, Monsell S, Driver J (2000). An intention-activation account of residual switch costs. Control of cognitive processes: Attention and performance XVIII.

[CR9] Desmet C, Fias W, Brass M (2012). Preparing or executing the wrong task: The influence on switch effects. The Quarterly Journal of Experimental Psychology.

[CR10] Duncan J, Burgess P, Emslie H (1995). Fluid intelligence after frontal-lobe lesions. Neuropsychologia.

[CR11] Kiesel A, Steinhauser M, Wendt M, Falkenstein M, Jost K, Philipp AM, Koch I (2010). Control and interference in task switching—A review. Psychological Bulletin.

[CR12] Lenartowicz A, Yeung N, Cohen JD (2011). No-go trials can modulate switch cost by interfering with effects of task preparation. Psychological Research.

[CR13] Los SA, Van der Burg E (2010). The origin of switch costs: Task preparation or task application?. Quarterly Journal of Experimental Psychology (2006).

[CR14] Meiran N (2000). Modeling cognitive control in task-switching. Psychological Research.

[CR15] Milner B (1963). Effects of different brain lesions on card sorting. Archives of Neurology.

[CR16] Monsell S (2003). Task switching. Trends in Cognitive Sciences.

[CR17] Monsell S, Mizon GA (2006). Can the task-cuing paradigm measure an endogenous task-set reconfiguration process?. Journal of Experimental Psychology: Human Perception and Performance.

[CR18] Morey, R. D., Rouder, J. N. (2018). BayesFactor: Computation of Bayes Factors for Common Designs. R package version 0.9.12-4.2. https://CRAN.R-project.org/package=BayesFactor.

[CR19] Oberauer K, Souza AS, Druey MD, Gade M (2013). Analogous mechanisms of selection and updating in declarative and procedural working memory: Experiments and a computational model. Cognitive Psychology.

[CR20] Rogers RD, Monsell S (1995). Costs of a predictable switch between simple cognitive tasks. Journal of Experimental Psychology: General.

[CR21] Rouder JN, Speckman PL, Sun D, Morey RD, Iverson G (2009). Bayesian *t* tests for accepting and rejecting the null hypothesis. Psychonomic Bulletin & Review.

[CR22] Rubinstein JS, Meyer DE, Evans JE (2001). Executive control of cognitive processes in task switching. Journal of Experimental Psychology: Human Perception and Performance.

[CR28] R Core Team (2013). R: A language and environment for statistical computing. R Foundation for Statistical Computing, Vienna, Austria. http://www.R-project.org/.

[CR23] Schneider DW, Logan GD (2005). Modeling task switching without switching tasks: A short-term priming account of explicitly cued performance. Journal of Experimental Psychology: General.

[CR24] Schuch S, Koch I (2003). The role of response selection for inhibition of task sets in task shifting. Journal of Experimental Psychology: Human Perception and Performance.

[CR25] Singmann, H., Bolker, B., Westfall, J., & Aust, F. (2017). afex: Analysis of Factorial Experiments. R package, version 0.18-0. https://CRAN.R-project.org/package=afex.

[CR26] Swainson R, Martin D (2013). Covert judgements are sufficient to trigger subsequent task-switching costs. Psychological Research.

[CR27] Swainson R, Martin D, Prosser L (2017). Task-switch costs subsequent to cue-only trials. Quarterly Journal of Experimental Psychology.

[CR29] Vandierendonck A, Liefooghe B, Verbruggen F (2010). Task switching: Interplay of reconfiguration and interference control. Psychological Bulletin.

[CR30] Verbruggen F, Liefooghe B, Szmalec A, Vandierendonck A (2005). Inhibiting responses when switching: Does it matter?. Experimental Psychology.

[CR31] Verbruggen F, Liefooghe B, Vandierendonck A (2006). Selective stopping in task switching: The role of response selection and response execution. Experimental Psychology.

[CR32] Verbruggen F, Liefooghe B, Vandierendonck A, Demanet J (2007). Short cue presentations encourage advance task preparation: A recipe to diminish the residual switch cost. Journal of Experimental Psychology. Learning, Memory, and Cognition.

[CR33] Verbruggen F, McAndrew A, Weidemann G, Stevens T, McLaren IPL (2016). Limits of executive control: Sequential effects in predictable environments. Psychological Science.

[CR34] Waszak F, Wenke D, Brass M (2008). Cross-talk of instructed and applied arbitrary visuomotor mappings. Acta Psychologica.

[CR35] Wickham H (2007). Reshaping data with the reshape package. Journal of Statistical Software.

[CR36] Wickham H (2009). ggplot2: Elegant Graphics for Data Analysis.

[CR37] Wickham, H. (2017). tidyr: Easily Tidy Data with ‘spread()’ and ‘gather()’ Functions. R package version 0.6.2. Retrieved from: https://CRAN.R-project.org/package=tidyr.

[CR38] Wickham, H., & Francois, R. (2016). dplyr: A Grammar of Data Manipulation. R package version 0.5.0. Retrieved from: https://CRAN.R-project.org/package=dplyr.

